# Characterization of African swine fever outbreaks in Hong Kong SAR, winter 2023 to 2024

**DOI:** 10.1128/spectrum.03663-25

**Published:** 2026-04-02

**Authors:** Lynnette C. Goatley, Laila Al-Adwani, Priscilla Y. L. Tng, Candy C. Y. Lau, Timothy T. L. Ng, Christopher J. Brackman, Martin Ashby, Carrie Batten, Karina W. S. Tam, Christopher L. Netherton

**Affiliations:** 1 The Pirbright Institute 111636, Woking, Surrey, United Kingdom; 2 Agriculture, Fisheries and Conservation Department, Government of the Hong Kong Special Administrative Region, Hong Kong, Hong Kong SAR, China; Erasmus MC, Rotterdam, the Netherlands

**Keywords:** African swine fever virus, genomics, veterinary pathogens, pathogenesis

## Abstract

Hong Kong veterinary authorities responded to a series of suspected outbreaks of African swine fever in domestic pigs in the New Territories of the Hong Kong Special Administrative Region from November 2023 to January 2024. Cases were identified on 10 farms, with one farm having a single pig with an inconclusive result. Subsequent genetic analysis suggested a more complicated picture than initially considered. Full genome sequencing identified a genotype II virus with a novel deletion in the left of the genome, as well as a genotype I/II hybrid virus similar to those previously reported in the Chinese mainland, Vietnam, and Russia. The replication kinetics of the two viruses were compared to reference strains *in vitro*, and the virulence of the genotype I/II hybrid was tested *in vivo* alongside an African genotype XV virus. Both viruses caused high fever and inappetence, with animals reaching severe humane endpoints within 6 days.

**IMPORTANCE:**

African swine fever virus causes a lethal hemorrhagic disease in domestic and wild pigs that is present in Africa, Asia, Europe, Oceania, and the Caribbean island of Hispaniola. The virus is a serious threat to pig farmers, global food security, and biodiversity. The virus has been circulating in Eurasia for nearly 20 years, and both small and large changes in the genome have been observed, including a new recombinant virus that emerged in 2021. Full genome sequencing of viruses is required to identify any changes that are important for understanding the epidemiology of the virus, as well as guiding vaccine design and selection. Here, we report genomic data from outbreaks in Hong Kong SAR during Winter 2023/2034 and evaluate the pathogenicity of a hybrid-recombinant virus. We show that minor changes to the hybrid-recombinant genome do not influence virulence and show that this virus, along with a previously uncharacterized isolate from Africa, can be used to test vaccine efficacy in future studies.

## INTRODUCTION

African swine fever (ASF) is a viral hemorrhagic fever that affects all species within the genus *Sus*, including domestic pigs and wild boar, and is nearly always fatal. The virus also infects other suids, but does not cause disease in bushpigs (*Potamochoerus larvatus*) or common warthogs (*Phacochoerus africanus*), while the susceptibility of babirusas and pygmy hogs is not fully known. As such, ASF presents both a serious threat to pig farmers, global food security, and biodiversity in southern and southeastern Asia, as well as Oceania, where a number of endangered suid species are currently extant (1–3).

ASF was first reported in Kenya in the early twentieth century with outbreaks in domestic pigs reported across southern and eastern Africa through the first half of the century. In 1957 and 1960, the virus was introduced into Portugal, becoming endemic on the Iberian Peninsula, with subsequent outbreaks across Europe, Brazil, and the Caribbean. With the exception of Sardinia, ASF was eradicated outside of Africa by the mid-1990s, but reappeared in 2007 when the virus was introduced to Georgia. ASF then spread across European Russia reaching Lithuania and Poland in 2014 and then into central Europe. The virus was reported in China for the first time in August 2018 and then spread across Asia and into Oceania.

African swine fever virus (ASFV) is a large double-stranded DNA virus with a genome size of between 170 and 193 kilobase pairs (kbp). ASFV is currently classified as *Asfivirus haemorrhagiae*, a species in the *Asfivirus* genus within the *Asfarviridae* family (4). The virus encodes for at least 150 major open reading frames (ORFs) (5), but recent transcriptional mapping has identified transcription start sites consistent with more than 300 ORFs (6, 7), suggesting that gene content may be more complex than initially thought. ASFV isolates have been typed through a number of different methods, mostly focusing on short sequences that can be reliably Sanger sequenced. Twenty-three genotypes (I to XVII, XIX to XXIV) are currently recognized based on approximately 400 bp of the 3′ end of the *B646L* gene, which encodes the major capsid protein p72 (8–13). The viruses introduced into Portugal in the twentieth century were 3′ *B646L* genotype I, whereas the prototype of the current panzootic, Georgia 2007/1 (GEO2007/1), is a genotype II virus (14, 15). A number of loci have been selected to subtype 3′ *B646L* genotypes with the most common being the central variable region (CVR) within the *B602L* gene (16, 17). CVR variants are reported based on the tetrapeptide repeats encoded by the *B602L* gene (16), and numerous variants have been identified in genotype I ASFV isolates (18, 19), but this region has proven remarkably stable in genotype II ASFV with only six major variants detected in Europe and Asia and three minor variants, with most viruses being identical to GEO2007/1 (19–21). Outside of Eurasia, a genetically distinct genotype II virus has caused recent outbreaks across Africa (22–25). Genotype I ASFV related to the old European strains circulates in Western Africa, and genotypes IX and X are regularly detected in central and eastern Africa (26–28). Genotypes I, II, and XIV have caused recent outbreaks in Zambia (29, 30), and genotype XV has been reported in both domestic animals and ticks in Tanzania (31–33).

After the initial outbreak of genotype II ASFV in China, low-virulent genotype I viruses emerged (34) that were similar to viruses previously reported in the field in Portugal (35, 36). Recombination between low-virulent genotype I and circulating genotype II ASFV generated a fully virulent genotype I/II hybrid virus (37), which has since spread to Vietnam and eastern Russia (38, 39). These viruses contain 10 sections from each parental strain, with the *B646L* and *B602L* gene derived from genotype I and the *E183L* and *EP402R* genes—two other genes commonly used for typing (40–42)—derived from genotype II. Recently licensed vaccines that are effective against genotype II ASFV do not protect against genotype I/II hybrids (37, 43), further complicating disease control in Asia.

ASFV was first reported in a domestic pig farm in the Hong Kong Special Administrative Region (Hong Kong SAR) in 2021 (44). Since then, ASFV has been detected in wild boar in several locations throughout the territory in 2021 and 2022 (45) and in an isolated domestic farm outbreak in February 2023 (https://wahis.woah.org/#/in-review/4898?reportId=159266).

Here, we present details focusing on the molecular epidemiological investigation into the outbreaks of ASFV in domestic pigs in Hong Kong SAR in the winter of 2023/2024. Full genome sequencing confirmed the presence of two genetically distinct viruses circulating at this time and compared the replication of these isolates *in vitro*. In addition, we characterized a virus consistent with the 3′ *B646L* genotype I/II hybrid recombinant alongside an historic genotype XV virus from Tanzania in domestic pigs.

## RESULTS

### Epidemiological investigation into African swine fever outbreaks in Hong Kong SAR

**Fig 1:**
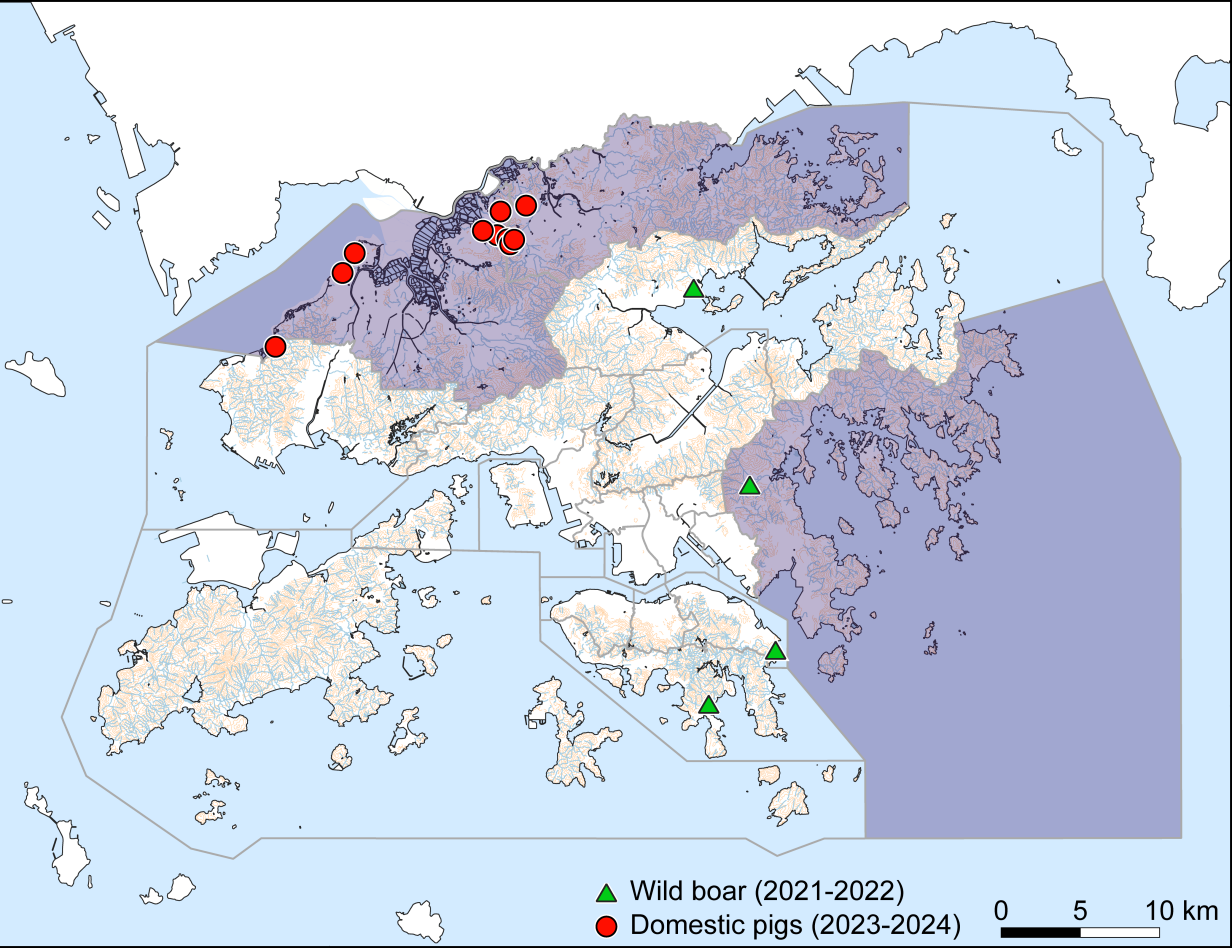
African swine fever outbreaks in Hong Kong SAR from 2021 to 2024. Geospatial data of Hong Kong SAR was downloaded from DATA.GOV.HK (accessed 14/01/2026), manipulated in QGis 3.22.4-Białowieża, and colored with Adobe Illustrator 29.7. Hydrological features are indicated in blue and contours in tan. Gray lines indicate boundaries between districts, and districts shaded purple contain pig farms. The locations of the cases of ASF in wild boar reported in 2021 and 2022 are shown as green triangles, and cases of ASF in domestic pigs in 2023 and 2024 as red circles.

From November 2023 to January 2024, a total of 10 pig farms in Hong Kong SAR were confirmed positive for ASFV. Affected farms were in the northern area of Hong Kong SAR in Yuen Long District and North District (Fig. 1). At the time, there were 43 pig farms in Hong Kong SAR, mostly concentrated in these districts as well as in Sai Kung District to the east. The following summarizes the outbreak investigations, clinical signs, and laboratory results from each farm in chronological order.

#### Farm 1: Lau Fau Shan (5 November 2023)

On 5 November 2023, the Agriculture, Fisheries and Conservation Department (AFCD) was notified by the farmer of Farm 1 in Lau Fau Shan about a suspected disease outbreak causing an increased mortality in sows. Ten sows were found dead over a period of 10 days. These sows had abortions, vomiting, and diarrhea. An inspection was conducted on the farm on 6 November 2023. Oronasal samples from 32 pigs, involving 30 sows in the breeder area and two growers housed nearby, were collected. Apart from a few sick sows with signs of dullness and abortion, no significant clinical signs were observed from other pigs in the farm. Oronasal samples from 16 sows tested positive for ASFV on 7 November 2023. As part of the disease control measures, the AFCD implemented temporary movement control to Farm 1, other subsequent ASF-infected farms, and surrounding farms within a 3 km radius.

#### Farm 2: Lau Fau Shan (22 November 2023)

Farm 2, which is approximately 1.5 km from Farm 1 in Lau Fau Shan, was inspected by the AFCD on 22 November 2023 after the farmer reported that some pigs were dead and others had shown clinical signs of inappetence and reddening of the skin for more than a week. Oronasal samples from three (out of 10) sows, one (out of 10) finisher, and one (out of 10) grower collected during the inspection tested positive for ASFV on 23 November 2023.

#### Farm 3: San Tin (8 December 2023)

The farmer of Farm 3 in San Tin reported a suspected ASF outbreak on 8 December 2023, as a few sows were found dead over a week and showed signs of reddening of the ears and skin, abortion, and bloody diarrhea. An inspection was conducted on the same day by the AFCD. Oronasal swabs from 10 (out of 10) sows, eight (out of 10) finishers, and one (out of 10) grower tested positive for ASFV on 9 December 2023.

#### Farm 4: Lau Fau Shan (22 December 2023)

The farmer of Farm 4 in Lau Fau Shan reported a suspected ASF outbreak on 22 December 2023, and an inspection was conducted on the same day by the AFCD. Oronasal samples from seven (out of 50) sows, zero (out of five) finishers, and zero (out of five) growers tested positive for ASFV.

#### Farm 5: San Tin (27 December 2023)

The farmer of Farm 5 in San Tin reported a suspected ASF outbreak on 27 December 2023. The pigs were generally inappetent, while some finishers died with mild nasal bleeding, and some sows died without any clinical signs other than inappetence over the past few days. An inspection was conducted on the same day by the AFCD. Oronasal samples from 20 (out of 25) pigs tested positive for ASFV.

As the number of farms infected with ASF increased rapidly during this period, at the end of December 2023, the AFCD began to distribute sampling materials to farmers for the collection of samples from pigs showing abnormalities to facilitate early ASF detection and responses.

#### Farm 6: San Tin (29 December 2023)

The farmer of Farm 6 in San Tin reported five dead sows with nasal and oral bleeding on 29 December 2023. Oronasal samples from five (out of five) dead sows and five (out of five) live pigs with clinical signs tested positive for ASFV.

#### Farm 7: San Tin (28 December 2023 to 1 January 2024)

No clinical signs in pigs at Farm 7 in San Tin were observed during an inspection by the AFCD on 28 December 2024. On 1 January 2024, the farmer collected oronasal samples from 13 dead pigs, of which eight pigs tested positive for ASFV.

#### Farm 8: Kwu Tung (28 December 2023 to 2 January 2024)

The inspection on 28 December 2023 revealed no clinical abnormalities in pigs at Farm 8 in Kwu Tung. An increased number of sick sows with inappetence and a higher abortion rate was noted later. On 2 January 2024, the farmer submitted six oronasal samples from three sick sows to the AFCD for ASFV testing. Results showed two out of the six samples were ASFV positive.

#### Farm 9: San Tin (28 December 2023 to 9 January 2024)

Multiple inspections and testing were conducted at Farm 9 in San Tin during the movement control period starting 28 December 2023. No significant abnormalities were noted, and ASFV test results were negative. An inspection was conducted on 9 January 2024, prior to lifting its movement control, and oronasal samples were collected from 30 live pigs and two dead pigs (one sow and one grower) for ASFV testing. The dead sow was found dead without any specific clinical signs, and the results showed the dead sow tested positive for ASFV.

#### Farm 10: San Tin (9–13 January 2024)

An inspection in Farm 10 in San Tin was conducted on 9 January 2024, during which oronasal samples from 30 pigs were collected, which tested negative for ASFV, and no abnormalities were noted on the general health conditions of the pigs. On 13 January 2024, the farmer reported that some pigs were inactive and collected oronasal samples from three sick pigs. All three samples tested positive for ASFV.

A summary of the outbreak data is provided in Table 1.

**TABLE 1:** Summary of ASF-affected pig farms in Hong Kong from November 2023 to January 2024

Farm	Location	Date	Number of pigs (positive/total)	3′ *B646L* genotype	Summary of clinical signs^*a*^
1	Lau Fau Shan	7 (Nov 2023)	16/32	II	Abortions, vomiting, and diarrhea were observed in sows prior to their death, along with dullness and abortion in some sick sows.
2	Lau Fau Shan	23 (Nov 2023)	5/30	II	Dead pigs (unspecified age group) with clinical signs of inappetence and reddening of skin for over a week.
3	San Tin	9 Dec 2023	19/30	II	Dead sows with reddening of ears and skin, abortion, and bloody diarrhea.
4	Lau Fau Shan	23 Dec 2023	7/60	II	A loss of appetite was noted in sows, accompanied by increased mortality.
5	San Tin	28 Dec 2023	20/25	II	Inappetence was noted; some finishers died with mild nasal bleeding, while some sows died without showing specific clinical signs other than inappetence.
6	San Tin	30 Dec 2023	10/10	II	Increased mortality among sows showing clinical signs of nasal and oral bleeding.
7	San Tin	2 Jan 2024	8/13	II	Increased mortality in sows and finishers with reddening of skin.
8	Kwu Tung	3 Jan 2024	2/3^*b*^	Hybrid	An increased number of sick sows with inappetence and a higher abortion rate.
9	San Tin	10 Jan 2024	1/32	II	A dead sow was found without specific clinical signs. No significant abnormalities were noted in other pigs.
10	San Tin	13 Jan 2024	3/3	II	No abnormalities, except a number of inactive pigs noted.

^*a*^ The description of the clinical signs on the farms is a combination of those described by the farmers and reported by AFCD and therefore should not be considered as a veterinary assessment.

^*b*^ Two samples each were collected from three pigs; however, the labelling was incomplete; therefore, it is not clear if the positive samples came from two pigs or a single animal.

For initial molecular characterization, blood samples from Farms 1 to 3 and oronasal swabs from Farms 4 to 10 were sequenced following real-time PCR confirmation. Genotyping based on the C-terminal region of the B646L gene identified the samples from nine farms as genotype II, while the sample from Farm 8 was identified as genotype I. Sequencing of the E183L gene classified all samples as genotype II. Further analysis of the EP402R gene showed that all samples clustered within serotype 8. To enable in-depth genomic analysis and comparison, virus isolation and high-throughput sequencing were performed on selected samples.

### Full genome sequencing of Hong Kong SAR outbreaks

Samples were subject to direct Illumina sequencing and virus isolation on primary bone marrow macrophage cultures. Hemadsorbing virus was successfully cultured from four sites—Farms 1, 3, 8, and 9—preliminary. Sanger sequencing confirmed that the virus from Farm 8 was 3′ *B646L* genotype I and also had an extended central variable region also consistent with genotype I ASFV. This sample, along with the virus isolated from Farm 3, was subject to limit dilution on bone marrow macrophage cultures before combined Nanopore and 300PE Illumina sequencing, along with those isolated from Farms 1 to 9. Reads generated from cultured virus and direct sequencing of clinical samples were assembled *de novo*. Briefly, clinical samples with Ct values ranging from 14.20 to 29.55 were subjected to direct sequencing on the Illumina NovaSeq 6000 (Table 2; Table S1). The average number of sequencing reads (read 1 and read 2) was approximately 188 million, except for Farm 4, which generated 2,243 million reads due to the need to repeat sequencing runs following an initial failure in genome assembly. The percentage of ASFV reads (read 1 and read 2) varied from 0.001% to 0.362% (Table S2). All three clinical samples with Ct values below 20 (Farm 1, Farm 2, and Farm 3) achieved complete genome recovery, attaining 100% breadth at ≥10 × coverage, exceeding the 99.8% cutoff suggested by Shi et al. (46). For clinical samples with Ct values between 20 and 25, three out of four samples (Farm 5, Farm 7, and Farm 10) exhibited complete or nearly complete genome recovery. Notably, the Farm 7 sample was classified as a near-complete genome assembly, demonstrating a breadth of ≥10× coverage at 99.96%. Nucleotide positions with a depth of coverage (DP) less than 10 were found at both ends (positions 1–13 and 184,467–184,478), with DP values of 8 to 9 observed in 78 nucleotide positions between 68,337 and 68,414. In contrast, clinical samples obtained from Farm 6 demonstrated a breadth of ≥10× coverage at 97.01%, supported by Nanopore amplicon sequencing. Conversely, the sample from Farm 4, with a Ct value of 29.55, posed significant challenges for complete genome recovery. The correlation between successful complete genome recovery and a Ct cutoff of <20 aligns with the threshold suggested by Shi et al. (46). In summary, near-complete genomes were successfully assembled from all of the sites apart from Farm 4, where only a partial genome could be assembled with a significant number of gaps (Table 1). Hybrid assemblies of virus cultured from Farms 1, 3, and 8 included sequences of the terminal inverted repeats, consistent with that previously identified by a PCR-Nanopore-based approach on the TLS/2019/1 isolate (47). Sequences obtained from clinical samples were practically identical to those obtained from cultured viruses; however, differences in the lengths of homopolymers at the left end of the genome were observed, which are typically difficult to resolve accurately (48). However, position 161,632 in the assembly of Farm 1 cultured virus was a mixed SNP (T 46.1%, A 26.4%, G 27.5%; coverage 967), whereas that derived from direct sequencing of the clinical sample was homogeneous for T (585-fold coverage). This would lead to a Q104H mutation in the pE199L protein that is involved in fusion of the internal envelope with endosome during virus entry (49); as such, this could represent an early adaptation to tissue culture.

**TABLE 2:** Summary of isolates and final genome assemblies

Farm	Isolate name	Illumina breadth (coverage >10×)	Nanopore data?	Assembly length (base pairs)	Accession numbers
1	HK/DP/2023/LFS-12807-34	99.98	Yes	185,922	PX277557
2	HK/DP/2023/LFS-13422-8	100	No	184,344	PX448603
3	HK/DP/2023/ST-14219-23	100	Yes	185,916	PX277558
4	HK/DP/2023/LFS-14508-46	74.75	No	179,508	PX448604
5	HK/DP/2023/ST-14651-3	100	No	184,340	PX448605
6	HK/DP/2023/ST-14735-2	96.49	Yes	183,854	PX448606
7	HK/DP/2024/ST-00009-13	99.96	No	184,478	PX448607
8	HK/DP/2024/KT-00117-2/16	100	Yes	187,527	PX277559
9	HK/DP/2024/ST-00662-2	100	Yes	183,813	PX277560
10	HK/DP/2024/ST-00566-2	100	No	184,339	PX448608

The sequences obtained from the farms, apart from Farm 8, were effectively identical, having minor differences in poly C/G tracts at the left end. These viruses were all 3′ *B646L* genotype II and shared the same CVR and I329L/I73R intergenic sequence as CHN/HLJ/2018 (MK333180.1). Compared to CHN/HLJ/2018 and GEO2007/1 (FR682468.2), the genotype II viruses had two deletions in the left end of the genome within the multigene family 110 (MGF110) region (Fig. 1A). A small deletion of 688 bp caused an in-frame fusion of the *MGF110-3L* and *MGF110-4L* genes. A larger deletion of 5,643 bp removed the *MGF110-7L*, *285L*, *MGF110-8L, 86R*, *MGF100-1R*, *MGF110-9L*, *MGF110-11L*, *MGF110-12L*, *MGF110-13L*, and *MGF110-14L* genes, as well as transcription start sites for five putative open reading frames: nORF_10297/10,302, nORF_12259, nORF_12624/12,626, nORF_12786, and ASFV_G_ACD_00240 (7). Comparison of the Farm 3 sequence to the original outbreak strain from China (HLJ/2018) revealed novel SNPs in the *MGF360-14L, B438L*, *G1340L*, *NP419L*, *D250R*, and *DP96R* genes (Table S3). Insertions and deletions were also observed in homopolymers in the genomes, particularly those in the left end. Insertions in a stretch of five Cs in the *MGF360-14L* gene led to a 70 aa C-terminal truncation, which had previously been reported in sequences from Korea, Vietnam, and Timor-Leste (47, 50).

**Fig 2:**
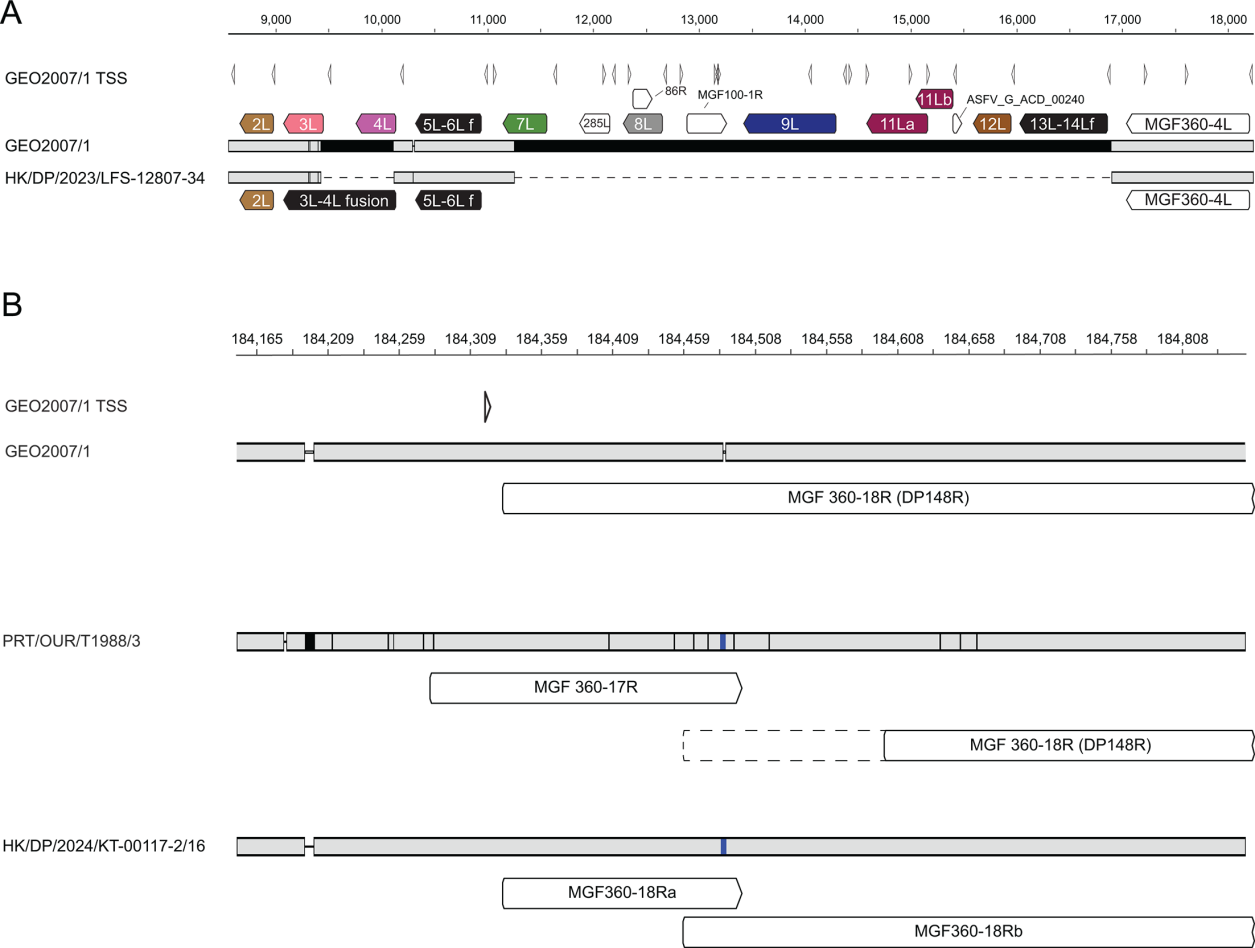
Differences between Hong Kong SAR viruses and reference sequences. (**A**) A deletion in the left end eliminates members of multigene family 110. Schematic of an alignment of GEO2007/1 and HK/DP/2023/LFS-12807-34 showing the approximate position relative to the GEO2007/1 genome, the transcription start sites (TSS) as mapped to the GEO2007/1 sequence, and the positions of the ORFs. Members of MGF110 are colored, and fusions are shown in black. (**B**) Alteration of the MGF360-18R (DP148R) open reading frame in HK/DP/2024/KT-00117-2/16. Schematic of an alignment between GEO2007/1, HK/DP/2024/KT-00117-2/16, and PRT/OUR/T1988/3. The position of a cytosine insertion within HK/DP/2024/KT-00117-2/16 relative to GEO2007/1 that is also present in PRT/OUR/T1988/3 is shown in as a blue bar. Annotated ORFs are indicated as open solid shapes below each sequence, with the unannotated part of MGF360-18R (DP148R) present in PRT/OUR/T1988/3 and PRT/NH/P68 outlined with dotted lines. (**A and B**) Identical regions of the genomes are shown in gray and differences in black, with deletions shown as dotted lines.

The genome sequence of HK/DP/2024/KT-00117-2/16 obtained from Farm 8 was very similar to the genotype I/II hybrid first identified on the in 2021 (CHN/JA/LG/2021, OQ504956.1). Novel SNPs and indels were identified in the *MGF505-5R*, *MGF505-6R*, *C257L*, *C962R*, *S273R*, and *MGF360-18R (DP148R*) genes (Table S4). In addition, differences within the central variable region of the *B602L* gene were identified (Table S4 and S5), one of which was novel and another which was shared with the hybrid recombinant identified in eastern Russia in 2023 (38). The tetrapeptide repeats encoded by the CVR of HK/DP/2024/KT-00117-2/16 represent the fifth different variant within the 11 hybrid recombinant genomes reported to date. The insertion of a cytosine within *MGF360-18R* leads to the premature truncation of the open reading frame immediately downstream of the transcription start site (Fig. 2B) and was therefore confirmed by Sanger sequencing. An alternative open reading frame, consistent with 10 codons fused to 186 codons of the C-terminus of pDP148R, is present within the HK/DP/2024/KT-00117-2/16 genome and was annotated as MGF360-18Rb, with the shorter upstream fragment annotated as MGF360-18Ra. The low-virulent genotype I isolates PRT/OUR/T1988/3 and PRT/NHP/68 also have an extra cytosine at the same locus, and in these viruses, MGF360-18Rb and MGF360-18Ra are annotated as MGF360-18R, or as part of MGF360-17R, respectively (Fig. 2B). Deletion of *DP148R* leads to attenuation of the virulent genotype I virus Benin1997/1 in pigs (51), but not virulent genotype II viruses (52, 53), and therefore, this disruption of the gene could alter the pathogenicity of HK/DP/2024/KT-00117-2/16.

**Fig 3:**
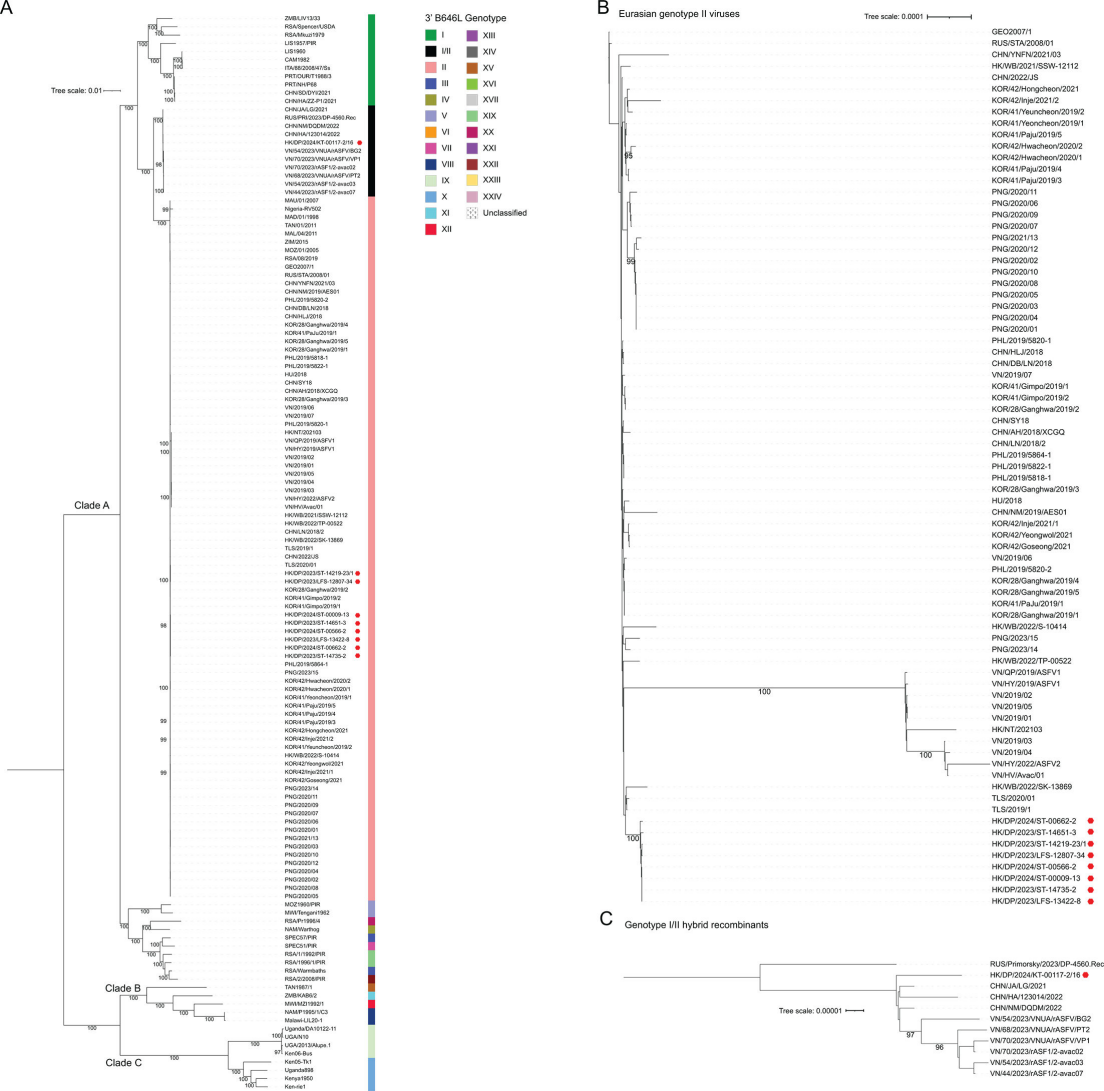
Genetic relatedness of African swine fever virus genome sequences inferred using the maximum likelihood method. Maximum likelihood phylogenetic trees were generated using IQ-TREE 3. The percentage of replicate trees in which the associated taxa clustered together in the ultrafast bootstrap test (10,000 replicates) in at least 95% of the trees is shown next to the branches. The 3′ *B646L* genotype for each sequence is indicated in the first column to the right of each tree. GTR + F + I + R4 (**A**) or HKY + F + I (**B and C**) models were selected using ModelFinder, and the trees were rooted at the mid-point (**A and C**) or on the GEO2007/1 sequence (**B**).

Full genome phylogeny placed the virus obtained from Farm 8 within the genotype I/II hybrids and those from the other farms with other genotype II viruses (Fig. 3). Most likely due to the large deletion in the MGF110 region of the Hong Kong SAR genotype II viruses, there was bootstrapping support for these viruses representing a separate cluster; however, there was no support for a relationship with other viruses from Hong Kong SAR or the wider region (Fig. 3B). Importantly, there was no genetic or geographic relationship (Fig. 1) between these viruses and those isolated from wild boar in 2021 and 2022 (45), arguing against a direct link between feral populations and the more recent outbreaks in domestic pigs. Although we cannot rule out continual evolution within wild populations, as observed in other regions, there is currently no evidence supporting this locally. Similarly, there was no support for a relationship between the hybrid recombinant isolated from Farm 8 and the hybrid recombinants from elsewhere, although the sequences from Vietnam do appear to cluster separately (Fig. 3C).

### Characterization of African swine fever viruses from Hong Kong and a historic genotype XV virus from Tanzania *in vitro* and *in vivo*

**Fig 4:**
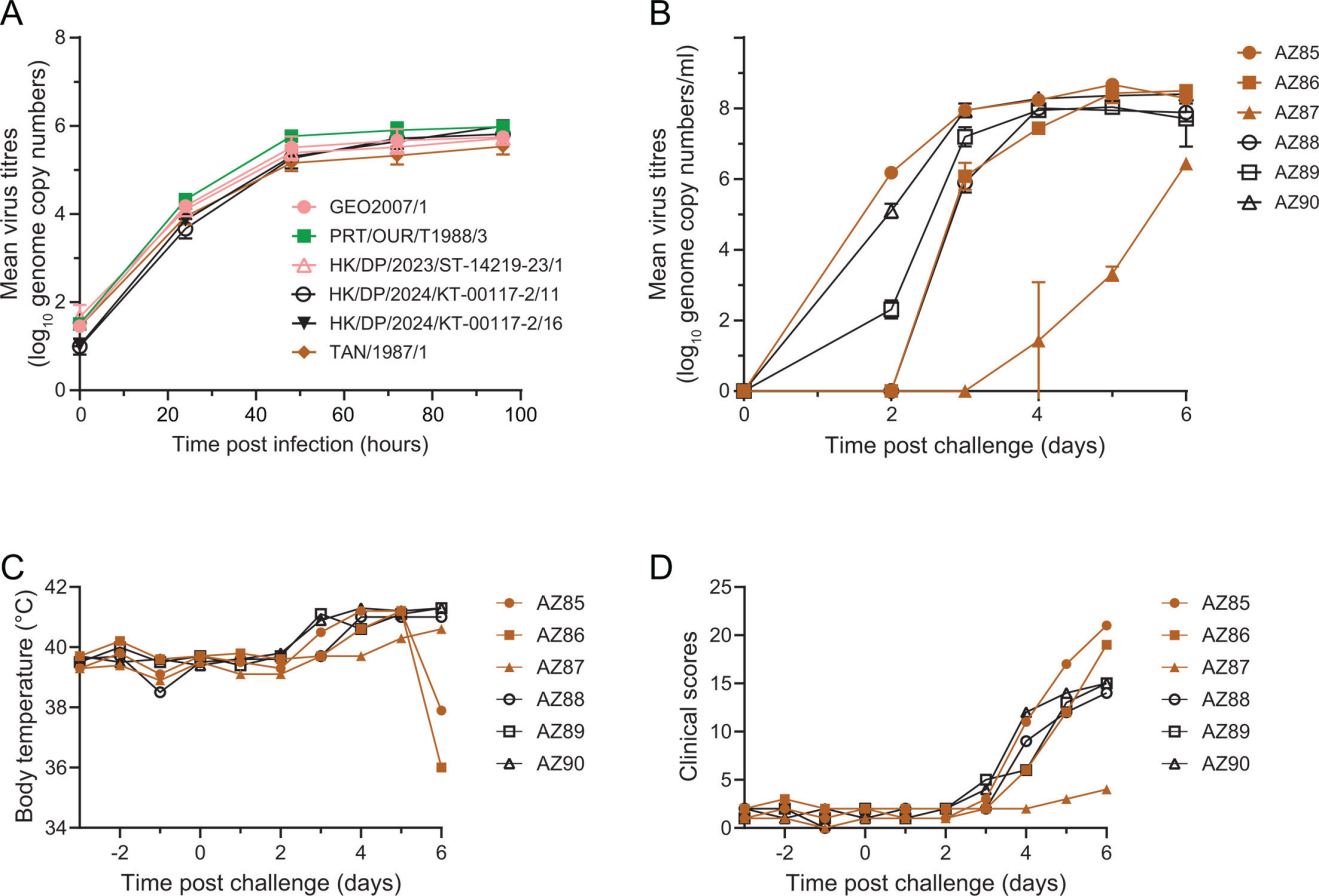
*In vitro* and *in vivo* replication. (**A**) Bone marrow-derived macrophage cultures were infected with the indicated viruses for 1 h at a multiplicity of infection of 0.01, and cells and supernatant were collected at the indicated times. Pigs were challenged with TAN1987/1 (brown) or HK/DP/2024/KT-00117-2/16 (black). Blood samples were collected on the indicated days to measure viremia (**B**), rectal temperatures (**C**), and clinical scores (**D**) were measured daily. Virus titers in samples were determined by qPCR (**A and B**).

Replication of viruses from Farms 3 and 8 was evaluated in primary macrophages and compared to the prototype genotype II virus GEO2007/1, the low-virulent field genotype I isolate PRT/OUR/T1988/3, or the Clade B genotype XV isolate TAN 1987/1 (Fig. 4A). No clear differences in the replication of the Farm 3 virus compared to GEO2007/1 were observed at any time point (*P* > 0.1818), consistent with previous work demonstrating that the *MGF110-5L-6L*, *285L*, *MGF100-1R*, and *MGF110-9L* genes are not essential for virus replication (54–57). The genotype I/II hybrid isolated from Farm 8 replicated as efficiently as GEO2007/1, PRT/OUR/T1988/3, and genotype XV TAN1987/1.

**Fig 5:**
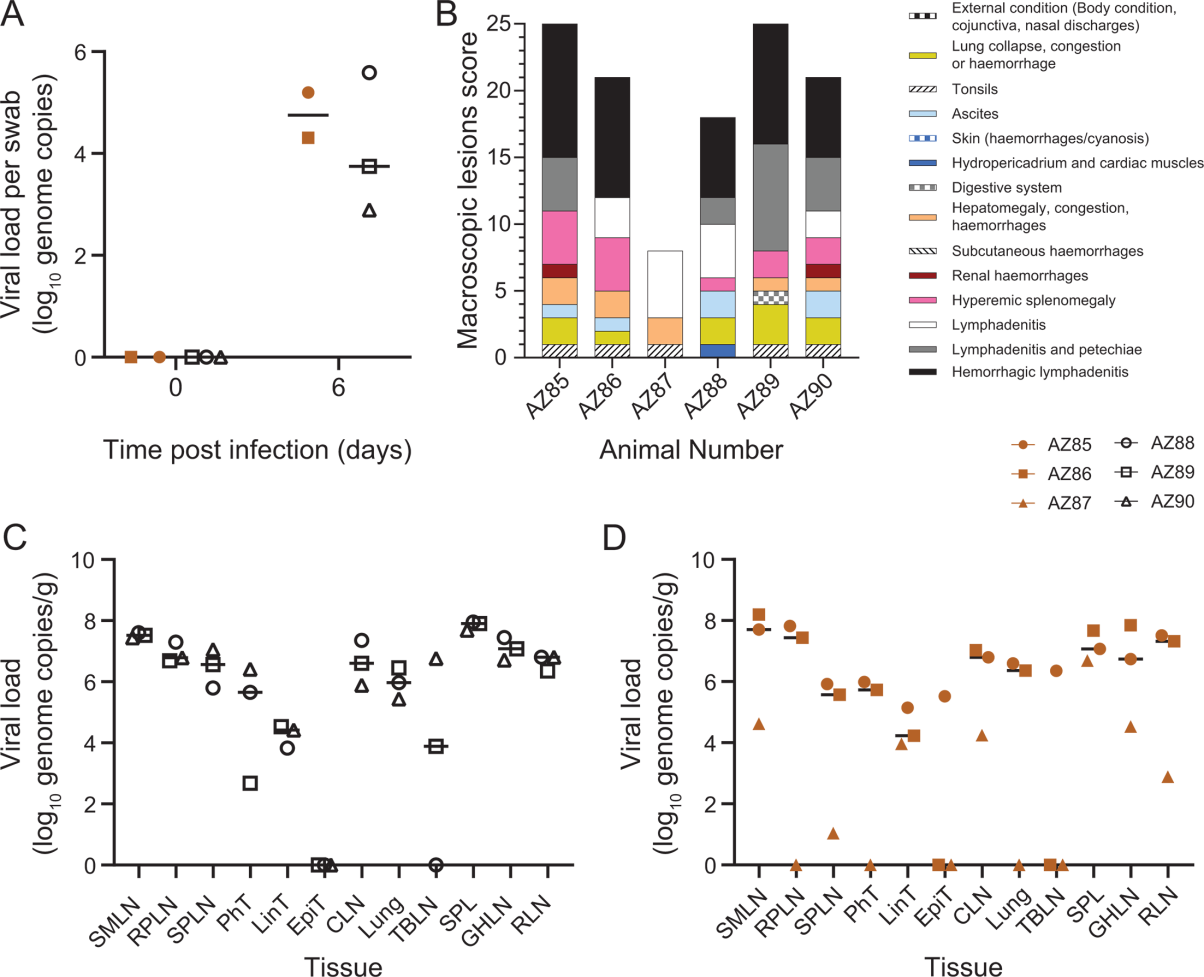
Assessment of macroscopic lesions and viral load in tissues. Samples from swabs collected before challenge or at termination (**A**), or the indicated tissues collected post-mortem from pigs infected with TAN1987/1 (**C**) or HK/DP/2024/KT-00117-2/16 (**D**), were homogenized, extracted, subject to qPCR, and then converted into viral load per gram of tissue. Data from submandibular lymph nodes (SMLN), retropharyngeal lymph nodes (RPLN), soft palate tonsil (SPT), pharyngeal tonsil (PhT), lingual tonsil (LinT), epiglottal tonsil (EpiT), cervical lymph node (CLN), lung, tracheobronchial (TBLN), spleen (SPL), gastrohepatic lymph node (GHLN), and renal lymph nodes (RLN) are indicated. Animals were assessed for macroscopic lesions during necropsies (**B**).

To assess the virulence of the Farm 8 virus, three pigs were inoculated by the intramuscular route with 1,000 hemadsorbing units of HK/DP/2024/KT-00117-2/16. To the best of our knowledge, the pathogenesis of genotype XV ASFV in an experimental setting has not been reported, and therefore, TAN1987/1 was also tested *in vivo* using the same route and dose. Two pigs infected with the Farm 8 virus first began to develop elevated temperatures 3 days post-infection (Fig. 4B), and all three pigs showed clinical signs consistent with acute ASF 4 days post-challenge (Fig. 4C). Six days post-infection, all three animals had refused food for three consecutive days and thus reached the humane endpoint (Table S6) and were euthanized. The pigs infected with TAN1987/1 showed a different clinical progression. Two pigs first showed elevated temperatures on the third and fourth days post-challenge and had both refused food on the fifth day. However, on the morning of the sixth day post-challenge, these two pigs were found moribund with body temperatures of 38°C or less and were euthanized. The third pig inoculated with TAN1987/1 had just begun to develop clinical disease on the fifth day post-challenge and was euthanized at the same time as the other animals to avoid lone housing of pigs. Necropsies revealed macroscopic lesions consistent with animals suffering acute ASF, with the exception of pig AZ87, which had a slower progression of disease (Fig. 5B). No obvious differences in the lesions were observed between the animals infected with HK/DP/2024/KT-00117-2/16 and TAN1987/1. Viremia closely followed clinical signs, with AZ88 infected with the Farm 8 virus having viremia of 6 log_10_ genome copies per mL of blood 3 days post-challenge and no clinical signs, whereas AZ89 and AZ90 had viremia of 7 and 8 log_10_, respectively, and both had high temperatures (Fig. 4D). Similarly, AZ87 had much lower viremia than the other two pigs infected with TAN1987/1. A virus was detected in nasal swabs from all of the animals that were tested at the end of the study (Fig. 5A). AZ85 and AZ86, which were found moribund 6 days post-challenge, had similar levels of viremia and viral load in the tissues when compared to the three animals infected with HK/DP/2024/KT-00117-2/16 (Fig. 5C and D). Although insufficient replicates were available to draw statistical significance, this does hint that viral replication alone was not responsible for the apparent differences in pathology of HK/DP/2024/KT-00117-2/16 and TAN1987/1. No obvious differences in hematological parameters were observed between the two groups of animals (Fig. S1).

## DISCUSSION

African swine fever has continued to threaten pig populations throughout Asia since its first identification in the Chinese mainland in August 2018, followed by rapid dissemination to multiple provinces. In Hong Kong SAR, the first outbreak in domestic pigs was reported in early 2021 (44), with subsequent ASFV detections in wild boar from various locations in 2021 and 2022 (45), and an isolated domestic farm outbreak in February 2023 (https://wahis.woah.org/#/in-review/4898?reportId=159266). All ASFV detected in Hong Kong SAR to date was highly virulent genotype II.

Since 2018, the majority of ASFV circulating in the Chinese mainland has been identified as highly virulent genotype II strain. A limited number of cases were reported to be caused by reduced-virulence variants of genotype II ASFV (58), genotype I ASFV (34) and, more recently, virulent genotype I/II hybrid ASFV (37). The genotype I/II hybrid virus has also been identified in nearby countries, including Vietnam and Russia (38, 39). The persistence of ASF outbreaks and the emergence of novel genotypes continue to present ongoing challenges for disease management. Understanding the epidemiology and genetic characteristics of ASFV circulating in Hong Kong SAR is essential for effective local control and prevention measures.

Genomic analysis of the samples demonstrated that nine of the outbreaks in Hong Kong SAR were caused by a genotype II virus with a novel deletion in the left end of the genome. This deletion led to the loss of seven members of MGF110, as well as the *MGF100-1R*, *285L*, and *86R* genes, and five other putative ORFs. Based on the standard Sanger sequencing genotyping assays, these viruses would be 3′ *B646L* genotype II, *E183L* genotype IIa, pB602L/CVR tandem repeat sequence class 10a, *I329L*/*I73R* intergenic region type 2, and pEP402R/CD2v serogroup 8, i.e., completely indistinguishable from the initial outbreak reported in China in 2018. Full genome sequencing enabled the identification of the deletions as well as eight novel SNPs and indels, along with potential differences in homopolymers. All of these differences were shared across eight farms that suffered outbreaks of genotype II ASFV, except for single SNPs within the *C475L* gene in Farm 5 and the *E199L* gene in the virus isolated from Farm 10. Although we only managed to generate a partial genome from Farm 4, this sequence also included the large deletion in the MGF110 region which was supported by 7-fold Illumina coverage. Therefore, it is highly likely that the outbreaks of genotype II reported in Hong Kong SAR in late 2023 and early 2024 were related; however, whether the virus spread from farm to farm or was linked in some other way is not known.

Phylogenetic analyses provided no clues as to the likely origin of the outbreaks in Hong Kong SAR, and regardless, accurate outbreak tracing of ASFV is often difficult due to the low genetic diversity and heterogeneous sampling efforts (59), the notable exception being in eastern Germany, where a frameshift in the *O174L* DNA polymerase X gene has likely led to an increased mutation rate (48). Nonetheless, the genotype II viruses appear to be distinct from other viruses isolated from Hong Kong SAR due to the absence, in the 2023/2024 viruses, of any unique SNPs found in these earlier sequences (44, 45). In addition, the complete deletion of MGF110-3L and the partial deletion of MGF110-4L found in HK/WB/2022/S-10414 are distinct from those observed in the genotype II viruses sequenced in this study (45).

The presence of the genotype I/II hybrid virus in a single farm suggested that there was no direct epidemiological link or transmission between this farm and others. It is likely, therefore, that there were two separate introductions of ASFV into domestic pigs in Hong Kong SAR in the winter of 2023/2024. Phylogenetic analyses also provided no clues on the source or the epidemiological linkage of this virus. The detection of a genotype I/II hybrid virus in Hong Kong SAR shows that this virus is continuing to circulate in the region, having been previously reported in Jiangsu, Henan, and Inner Mongolia in the Chinese ainland, four provinces across Vietnam, and Primorsky Krai in Eastern Russia. Analysis of the genome isolated from Farm 8 revealed a number of novel SNPs that have not yet been identified in the genomes of other hybrids. Considering the relatively few changes observed in the *B602L* CVR region in genotype II ASFV, it is striking that five different variants have been observed across the 11 genotype I/II genomes sequenced to date. The *B602L* gene from the hybrid recombinant came from the genotype I parent, and the genotype I CVR appears to be much more prone to change, with 21 variants recovered from pigs in Nigeria alone (60). Considering the essential role of pB602L in the recruitment of the major capsid protein p72 to viral membranes (61, 62), it is intriguing that relatively minor changes have been observed in the genotype II sequence over the last 20 years, but that genotype I ASFV can tolerate significant differences in CVR sequence lengths.

The differences between the hybrid genome found in Hong Kong SAR and the other genotype I/II genomes led us to test the pathogenicity of this virus in domestic pigs. Disease progression, viremia, and viral load in tissues of the genotype I/II hybrid HK/DP/2024/KT-00117-2/16 were comparable to that we have previously seen in pigs intramuscularly infected with virulent genotype I PRT/OUR/T1988/1, BEN1997/1, and genotype II GEO2007/1 (36, 63–66). Our data suggests that fragmentation of the MGF360-18R/DP148R ORF does not affect the virulence of the genotype I/II hybrid virus, which is similar to what has been reported for genotype II ASFV (52, 53). Genotype XV TAN1987/1 appeared to present differing pathology. Of note, neither AZ86 nor AZ87, which were found moribund 6 days post-infection with TAN1987/1, had reached moderate humane endpoints (65) 5 days post-challenge. This suggests that there was a rapid progression of disease between 5 and 6 days post-challenge. However, drawing firm conclusions about the virulence of different ASFV isolates is not possible with such small numbers of animals, and a more detailed study measuring other parameters, such as cytokine levels as well as histological changes, would be required to gain a better understanding of any differences in, or potential mechanisms of, pathology.

The continued circulation and spread of the genotype I/II hybrid virus are of particular concern, as none of the currently licensed genotype II vaccines offer effective protection against it (37, 43). Deployment of vaccines that are not effective against all circulating viruses within a region poses the risk of undermining confidence in ASF vaccines of any type. New approaches to vaccination are critically important, as it may be unrealistic to develop safe MLV vaccines with sufficient rapidity to combat an evolving virus. Nonetheless, this study establishes the pathogenicity in UK pigs of two circulating African swine virus isolates, enabling their use in future vaccination and challenge studies.

## MATERIALS AND METHODS

### ASFV diagnosis

DNA was extracted from blood samples and oronasal swab using NucliSENS easyMAG (bioMerieux, France), according to the manufacturer’s instructions. Real-time PCR was performed using the primers targeted the N-terminal conserved region of the B646L gene (forward: 5′-CTGCTCATGGTATCAATCTTATCGA-3′; reverse: 5′-GATACCACAAGATCRGCCGT-3′; probe: 5′-FAM-CCACGGGAGGAATACCAACCCAGTG-BHQ1-3′), as previously described.

### Viruses and cells

Virus cultures of, and animal experiments with, African swine fever virus were carried out in the high-containment laboratories at the Pirbright Institute in the United Kingdom of Great Britain and Northern Ireland, under a license granted by the Health and Safety Executive under Article 4(1) of the Specified Animal Pathogens Order (SAPO) 2008 (statutory instrument 2008/944). The GEO2007/1, PRT/OUR/T1988/3, and TAN1987/1 isolates have been described previously (14, 36, 67). Viruses were isolated, cultured, and titrated by endpoint dilution on porcine bone marrow–derived macrophages as described previously (13, 68). Viruses for combined Illumina and Nanopore sequencing were amplified on macrophages cultured from porcine bone marrow cells, which were purified by density gradient centrifugation over Histopaque 1083 (1,000 × *g*, 20 min, room temperature, no brake) and then cultured for 3 days in the presence of 100 ng/mL CSF-1 (Roslin Technologies). The virus was harvested after at least 90% cytopathic effect was observed, concentrated by ultracentrifugation, and DNA was extracted as previously described (13, 69).

### Sequencing and assembly

#### Direct sequencing and assembly from clinical samples

Libraries for Illumina sequencing were prepared using the Illumina Nextera XT protocol, with a modification of the amplification cycles set to 18. Sequencing was performed at the Centre for PanorOmic Sciences (CPOS), LKS Faculty of Medicine, the University of Hong Kong, on an Illumina NovaSeq 6000 platform using a 150 bp paired-end configuration. For the clinical sample obtained from Farm 6 (HK/DP/2023/ST-14735-2), additional amplicon sequencing was performed to confirm the genomic region with zero depth of coverage using the primer sets listed in Table 3. This was conducted following the official protocols of the native barcoding kit (SQK-NBD114.24) for MinION sequencing on a MinION MK 1C device with an R10.4.1 flow cell.

**TABLE 3:** Primers used for Sanger sequencing

Primer name	Primer sequence
L14L For	5′-CATAACATGGGTGGGAAACAAA
DP71L Rev	5′-GCATGTCCATTTTGCCACAG
DP148R Rev	5′-TGCCTCCCATCAAGTAAGCA
DP148R For	5′-ATGAGGAACCGGACTTTGCT
ASFV_Georgia_73549_F	5′-GTGAAAAGCTGATGGAAACC
ASFV_Georgia_75225_R	5′-GAGGGACGCATGTAGTAAAT
ASFV_Georgia_16630_F	5′-CAGGCAAGAAACATCATGAC
ASFV_Georgia_18208_R	5′-TGAGAGACAATTTGCGGTAA

Adapter sequences were removed, and Illumina sequencing reads with a length less than 100 bases or with a quality score below 10 were filtered out using BBDuk (BBMap version 37.62) (70). The remaining reads were subsequently mapped to the ASFV reference genomes using the Burrows–Wheeler Aligner (version 0.7.17) (71) and Samtools (version 2.0.4) (72). Draft assemblies were constructed from the mapped reads utilizing SPAdes (version 3.12.0) (73). For the clinical sample obtained from Farm 4 (HK/DP/2023/LFS-14508-46), the assembly process was conducted in accordance with the recommendations outlined by Spinard et al. (74). Briefly, contigs were compared against published ASFV genomes using BLASTplus (version 2.15.0) (75), and the top ASFV genome was selected as the reference for draft assembly. Contigs were mapped to this reference using Minimap2 (version 2.17) with the --asm5 option (76), and variant was applied to the reference as the first draft assembly, followed by a manual comparison of large insertions or deletions between the contigs and the draft assembly. Nucleotides in uncovered regions were designated as “N.”

Filtered Illumina sequencing reads were then mapped to the draft assembly, followed by variant calling using FreeBayes (version 1.3.10) for error correction (77), with visualization assisted by the Integrated Genome Viewer (IGV, version 2.16.0) (78). The breadth of coverage at 10× depth was calculated using Samtools (version 2.0.4) and an in-house Python script (Python version 3.11.5).

#### Sequencing and assembly after virus isolation

Libraries for Illumina and Nanopore sequencing were prepared as previously described (13), except that 2 µg of DNA was sheared using T7 endonuclease (NEB, M0302) for 30 min at 37°C prior to barcoding. Individual barcoding was performed using a native barcoding kit (SQK-NBD114-24) for MinION (MIN-101B) sequencing on a R10.4.1 MinION flow cell (FLO-MIN114), following the manufacturer’s instructions.

Trim Galore (0.6.10) was used to remove adaptor sequences and to select Illumina reads with a quality score >29. Chopper (0.5.0) and Porechop (0.2.4) were used to select Nanopore reads with a quality score >9 and length >299 bp. Hybrid assemblies were then performed using Spades (3.15.3) with the --isolate option (73). Nanopore-only assemblies were performed with Flye (2.9.2-b1786) using --genome-size 200k and --meta options, followed by one round of Medaka polishing (1.8.0) using the trimmed reads as input. Nanopore contigs were then aligned to the hybrid assemblies using Geneious Prime (2023.2.1) to identify contigs that extended the hybrid assemblies into the terminal inverted repeats. Finally, trimmed Nanopore and Illumina reads were mapped to correct for assembly errors and identify single nucleotide polymorphisms (SNPs). Assemblies were trimmed to remove any sections with <10-fold Illumina coverage, except for HK/DP/2023/LFS-12807-34, where a 29 bp stretch with a minimum 4-fold coverage was tolerated due to 150- to 157-fold Nanopore coverage.

DP148R sequence was confirmed by Sanger sequencing, as previously described (16), using a BigDye terminator v3.1 cycle sequencing kit (Applied Biosystems) and run on a 48-capillary ABI 3730 DNA analyzer using the primers indicated in Table 3. The region containing DP148R was amplified using three primer sets to generate overlapping amplicons: L14L For and DP71L Rev, L14L For and DP148R Rev, and DP148R For and DP71L Rev.

### Annotation

Open reading frames were identified by comparison with appropriate reference isolates using Geneious Prime v2025.1.3 (GraphPad Software, LLC), and multigene family members were assigned using the Imbrey nomenclature (79). The 3′ *B646L* genotype of the sequences was assigned based on previously published data (8, 10, 11). Open reading frames for the *KP177R*, *C105R*, *F165R*, *CP204L*, *CP312R,* and *G1207R/G1211R* genes were annotated as per their transcription start sites (6, 7, 59, 80). To aid clarity, copies of DP60R present at the left end of the genome were renamed as KP60L to maintain consistency with the annotation of genotype I and I/II hybrid recombinant viruses (5) and to better reflect the orientation of the gene. Regions in the assemblies of HK/DP/2023/ST-14735-2, HK/DP/2024/ST-00009-13, and HK/DP/2023/LFS-14508-46 with <10× fold Illumina coverage were highlighted. Annotations were converted to five-column feature files for upload to public repositories using GB2sequin (81).

### Alignment and phylogeny

PolyC/G tracts at the 5′ end of the genome, which are a common source of sequencing errors, were removed. These are located at positions 14,225–14,237, 15,666–15,682, 17,623–17,632, 17,838–17,846, 19,792–19,799, 19,993–20,008, and 21,797–21,802 relative to the Georgia 2007/1 sequence (FR682468.2), as previously described (48). The TIRs and extreme 5′ and 3′ ends were also removed (1 to 1,391 and 189,207 to 190,584 of FR682468.2). Genome sequences (Table S1) were aligned using Mafft v7.526 with iterative refinement, then manually corrected using Geneious Primer (v2025.1.3). Phylogeny was performed using IQ-TREE (v3.0.1) (82), with Modelfinder (83) and ultrafast bootstrapping (84). Trees were displayed using the Interactive Tree of Life (85), labeled with a modified version of the coloring scheme introduced by Mulumba-Mfumu et al. (86) and edited in Adobe Illustrator.

### Animal studies

All of the animal experiments were carried out under the Home Office Animals (Scientific Procedures) Act (1986) and were approved by the Animal Welfare and Ethical Review Board of the Pirbright Institute. The animals were housed in accordance with the Code of Practice for the Housing and Care of Animals Bred, Supplied, or Used for Scientific Purposes, and bedding and species-specific enrichment were provided throughout the study to ensure high standards of welfare. Through careful monitoring, pigs that reached the scientific or humane endpoints of the studies were euthanized by an overdose of anesthetic. All procedures were conducted by Personal License holders under the auspices of Project License PP8739708.

Six female Landrace × large white × Hampshire pigs were obtained from a high health farm in the UK and randomly assigned to each group prior to immunization. Piglets had been vaccinated against PCV2 (subunit), and sows and gilts were vaccinated against *E. coli* (multiple subunits of adhesins and a toxin), Erysipelas (inactivated), and Parvovirus (inactivated). The farm has been PRRSV-free since at least 2019. As the study was a descriptive study designed to test the pathogenesis of two different ASFV isolates, animal technicians and scientists were not blinded as to which group was which, nor was the number of animals determined by power calculations. Scoring of clinical signs and macroscopic lesions assessed at post-mortem was as described (59, 63, 87), with the exception that a score of 6 was given to animals with a body temperature less than 38°C. This changed the clinical scoring for temperature described in King et al. (63) to: 38 ≤ *x* < 39 = 0, 39.0 ≤ *x* < 39.5 = 1, 39.5 ≤ *x* < 40 = 2, 40.0 ≤ *x* < 40.6 = 3, 40.6 ≤ *x* ≤ 41 = 4, *x* > 41 = 5, *x* < 38 = 6, where *x* was the body temperature of the pig in degrees Celsius. Hematological measurements were taken from blood collected in EDTA using an IDEXX ProCyte Dx Analyzer (Model No: 98-70000-03) and ProCyte Dx Reagent Kit (Product: 99-26306-00), following the manufacturer’s instructions.

### ASFV genome quantification

DNA was extracted from tissue culture and whole blood samples in duplicate using the MagMAX CORE Nucleic Acid Purification Kit (ThermoFisher) on a Kingfisher Flex Extraction System. Tissues were homogenized using lysing matrix A tubes (MP Biomedicals) on beadbug machine (Sigma), and the DNA was extracted using MagMAX CORE Nucleic Acid Purification Kit. Quantitative PCR was carried out using the King assay (88) with qPCR Brilliant III Probe Master Mix with ROX (Agilent, 600890) on an AriaMx real-time PCR system (Agilent).

## Data Availability

All raw data is included in the text, figures, or Table S5. Raw sequencing data is available at the Sequence Read Archive under BioProject PRJNA1314468, and final assembled genomes have been assigned accession numbers PX277557–PX277560 and PX448603–PX448608.

## SUPPLEMENTAL MATERIAL

Figure S1 spectrum.03663-25-s0001.pdf Hematological changes. Crude blood cell population counts were determined using an IDEXX ProCyte Dx Analyzer. Each pig is plotted as a separate data point, and the lines indicate the mean of the group. Samples were not collected from pigs six days after infection with TAN1987/1 due to the moribund state of two of the animals.

Supplemental tables spectrum.03663-25-s0002.xlsx Tables S1 to S6.

ASM does not own the copyrights to Supplemental Material that may be linked to, or accessed through, an article. The authors have granted ASM a non-exclusive, world-wide license to publish the Supplemental Material files. Please contact the corresponding author directly for reuse.
