# Supplementary material for: Characterization of African swine fever outbreaks in Hong Kong SAR, winter 2023 to 2024
Source: Microbiol Spectr. 2026 Apr 2;14(5):e03663-25. doi: 10.1128/spectrum.03663-25 (PMC13141970; doi:10.1128/spectrum.03663-25)
Supplement: Figure S1 — Hematological changes. Crude blood cell population counts were determined using an IDEXX ProCyte Dx Analyzer. Each pig is plotted as a separate data point, and the lines indicate the mean of the group. Samples were not collected from pigs six days after infection with TAN1987/1 due to the moribund state of two of the animals. [file spectrum.03663-25-s0001.pdf]

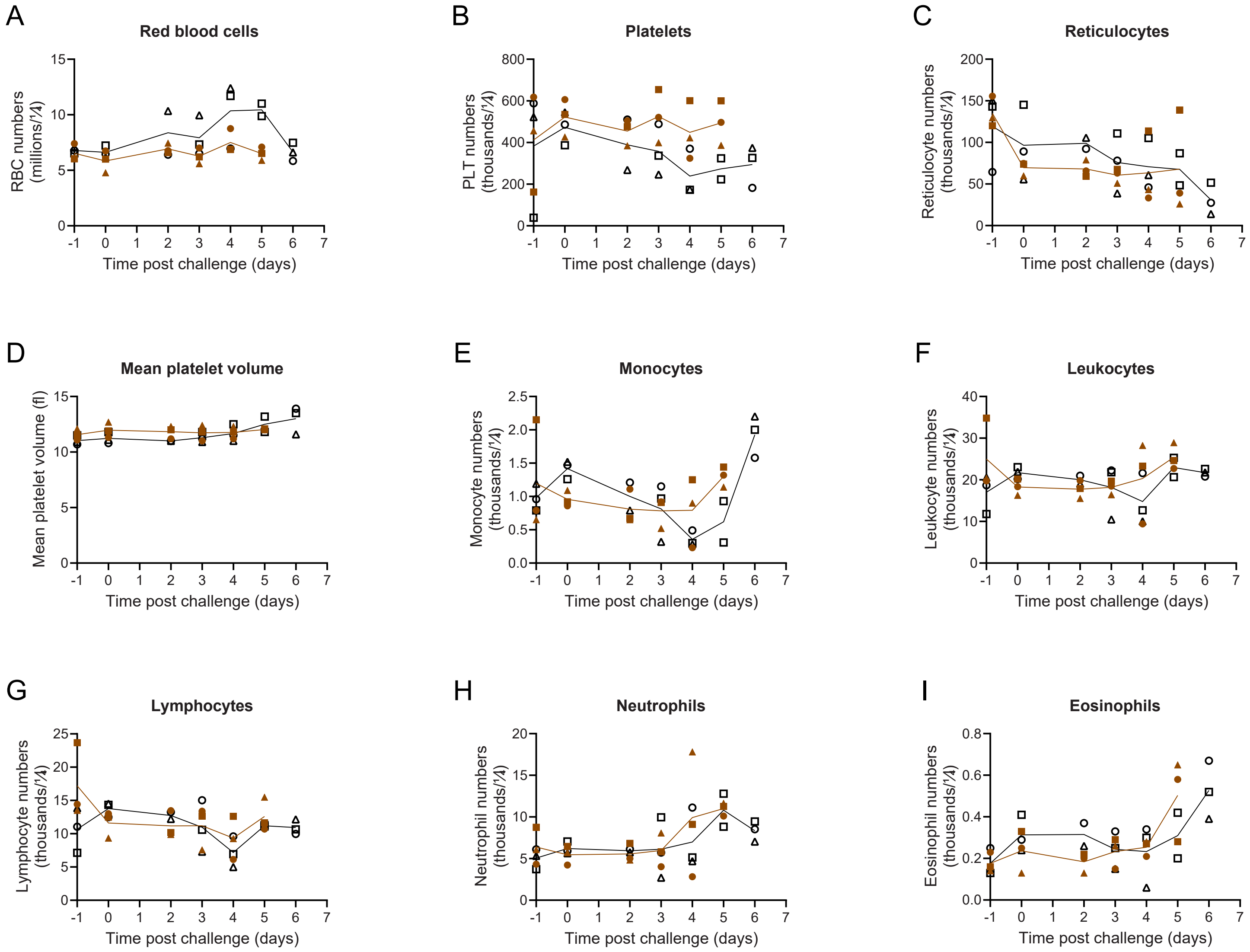


Supplementary Figure 1: Haematological changes. Crude blood cell population counts were determined using an IDEXX ProCyte Dx Analyser. Each pig is plotted as a separate data point and the lines indicate the mean of the group. Samples were not collected from pigs six days after infection with TAN1987/1 due to the moribund state of two of the animals.

AZ85
AZ86
AZ87
AZ88
AZ89
AZ90
